# Academic harassment: The need for interdependent actions of stakeholders

**DOI:** 10.1016/j.eclinm.2022.101481

**Published:** 2022-06-03

**Authors:** Susanne Täuber, Loraleigh Keashly, Sherry Moss, Jennifer Swann, Leah Hollis, Linda Crockett, Pooya Sareh, Morteza Mahmoudi

**Affiliations:** a Department of Human Resource Management & Organizational Behavior, University of Groningen, Groningen, the Netherlands; b Department of Communication, Wayne State University, MI, USA; c School of Business, Wake Forest University, NC, USA; d Department of Biological Sciences, Lehigh University, Bethlehem, PA, USA; e Department of Advanced Studies, Leadership and Policy, Morgan State University, Baltimore, MD, USA; f The Canadian Institute of Workplace Bullying and Harassment Resource Centre Inc., Alberta, Canada; g Creative Design Engineering Lab (Cdel), Department of Mechanical, Materials, and Aerospace Engineering, School of Engineering, University of Liverpool, Liverpool L69 3GH, UK; h Department of Radiology and Precision Health Program, Michigan State University, East Lansing, MI, USA

Scientific stakeholders have a critical role in addressing academic harassment, defined as the repeated acts of discrimination, incivility, or various types of harassment and threats against a target, in a timely and effective manner. However, their limited coordinated actions created a safe environment for bullies to thrive in academia.^1^ Therefore, creating platforms including conferences^2^ to bring various stakeholders together to share their insights on addressing academic bullying is critical, as it seems an effective pathway to remove “power poisoning” at any level in our science backyard,^1^ As scholars of academic harassment, we propose the following steps in improving the crosstalk between stakeholders

*Everyone can be an ally.* Many academics, in all disciplines and levels of experience, have witnessed and/or experienced academic harassment.^3^ Surveys that contain targets’ narratives (e.g.,^3^) reveals the devastating impact that bullies have on those they target, the pain they inflict, their harrowing effects psychological and physical health as well as on academic careers of targets. Everyone can be an ally by raising awareness about the academic harassment issue, and the urgent need in making academia a safe place for everyone.^4^ Therefore, targets, bystanders, survivors, and supporters of academic harassment should be more proactive and report incidences of harassment at least to any trustable and independent resources such as funding agencies and institutional ombuds offices.

*Raising awareness is key.* It is incredibly valuable for targets, but also for more powerful stakeholders, to understand the systemic and structural roots of academic harassment regardless of discipline or location. A solid policy to prevent bullying is a great first step.^5^ The insidious nuances, gaslighting and isolation involved in academic harassment can lead to a variety of medical diagnoses from adjustment disorder, depression, anxiety, symptoms of trauma, post-traumatic stress disorder (PTSD) and/or complex PTSD, which affirms what these targeted human beings are experiencing is valid. Any members of the scientific community can contribute to increasing awareness through various channels.^4^

*Funders lead the good fight.* Funding agencies can enforce anti-harassment policies. We believe a crucial step now is that funders unite, and exchange lessons learned and global best practices. For instance, the US National Institute of Health (NIH) shows that enforcement is key, and resources are essential to safeguard efficient enforcement (details on the NIH harassment and bullying resources are available at the NIH website^6^). In Europe, the European Commission just made gender equality plans mandatory for universities to be eligible for funding. The UK Wellcome Trust funding source also has policies for addressing academic bullying by sanctioning perpetrators and their institutions.^7^ We believe a crucial step now is that funders unite, and exchange lessons learned and global best practices.

*Bullying can be a career tool.* Recent developments and our increased understanding of academic bullying as a systemic problem have led to new insights^8^ including that academic bullying can be used as a career tool by “the mean and the mediocre”^8^ to climb the academic ladder more successfully than their targets. That is, academic bullying can be a strategic choice to get rid of competition, suggesting that the most talented scholars may be at the highest risk of being bullied. The implications for science and society are clear and sobering: how many great inventions, insights, and solutions have we missed by putting down and pushing out up and coming scholars? To solve this critical issue, at least in part, academic leaders should be selected among those “who are aware of the dynamics of power and privilege, and who will not incentivize bullying but rather stimulate community spirit”.^8^

*Make complaint procedures effective and safe*. Despite higher education institutions’ stated commitment to ensuring campuses are free from academic bullying, converging evidence suggests that the structures designed to provide care are unsafe themselves.^9^ Current institutional approaches to reporting commonly fail to acknowledge that the power asymmetries that enable academic harassment also apply to complaint procedures, undermining their effectiveness. In addition, some solutions offered to targets of academic harassment are well-intended but still costly for the target. We have recently argued that removing targets of academic bullying from their labs takes them out of the reach of their bully, but can have devastating effects on their careers.^9^

*Trauma experts and legal resources/support are needed*. We understand increasingly well that academic harassment can cause complex trauma and PTSD. The domino effect of the psychological injury can lead to the deterioration of mental and physical health and, ultimately, death. The effects of academic bullying are made worse by unsafe and inadequate institutional responses, which are often perceived as institutional betraya.^10^ In general, it appears that targets of academic bullying must finance their own legal response to document harassment experiences and secure damages – while the perpetrator, who is often in a management or leadership position, receives legal aid from the higher education institution. Stated differently, public and student money is used to maintain and perpetuate academic harassment. This practice is in urgent need of reform.

In summary, the next essential step for the scientific workforce should be to create a proactive collaborative platform among stakeholders who have an interest in and can influence academic harassment. All members of the scientific community are “responsible” and “response able” in creation and implementation of such proactive collaboration of stakeholders to make our scientific and disciplinary cultures safer, healthier, and more robust and effective.

## Contributors

All authors wrote and conceived the manuscript.

## Declaration of interests

Morteza Mahmoudi discloses that hScone is a co-founder and director of the Academic Parity Movement (www.paritymovement.org), a non-profit organization dedicated to addressing academic discrimination, violence, and incivility. Susanne Täuber, Loraleigh Keashly, Sherry Moss, Jennifer Swann, Leah Hollis, Linda Crockett, and Pooya Sareh discloses that they are advisors of the Academic Parity Movement.
